# Sirtuins and Gut Microbiota: Dynamics in Health and a Journey from Metabolic Dysfunction to Hepatocellular Carcinoma

**DOI:** 10.3390/cells14060466

**Published:** 2025-03-20

**Authors:** Mahmoud Zhra, Muhammad Affan Elahi, Aamira Tariq, Ahmed Abu-Zaid, Ahmed Yaqinuddin

**Affiliations:** 1 Department of Anatomy and Genetics, College of Medicine, Alfaisal University, Riyadh 11533, Saudi Arabia; mzahra@alfaisal.edu; 2 Department of Biochemistry and Molecular Medicine, College of Medicine, Alfaisal University, Riyadh 11533, Saudi Arabia; melahi@alfaisal.edu (M.A.E.); amabuzaid@alfaisal.edu (A.A.-Z.); 3 Department of Biosciences, COMSATS University Islamabad, Islamabad Campus, Islamabad 45550, Pakistan

**Keywords:** sirtuins (SIRTs), gut microbiota, metabolic dysfunction, hepatocellular carcinoma (HCC), non-alcoholic fatty liver disease (NAFLD)

## Abstract

Metabolic dysfunction leading to non-alcoholic fatty liver disease (NAFLD) exhibits distinct molecular and immune signatures that are influenced by factors like gut microbiota. The gut microbiome interacts with the liver via a bidirectional relationship with the gut–liver axis. Microbial metabolites, sirtuins, and immune responses are pivotal in different metabolic diseases. This extensive review explores the complex and multifaceted interrelationship between sirtuins and gut microbiota, highlighting their importance in health and disease, particularly metabolic dysfunction and hepatocellular carcinoma (HCC). Sirtuins (SIRTs), classified as a group of NAD^+^-dependent deacetylases, serve as crucial modulators of a wide spectrum of cellular functions, including metabolic pathways, the inflammatory response, and the process of senescence. Their subcellular localization and diverse functions link them to various health conditions, including NAFLD and cancer. Concurrently, the gut microbiota, comprising diverse microorganisms, significantly influences host metabolism and immune responses. Recent findings indicate that sirtuins modulate gut microbiota composition and function, while the microbiota can affect sirtuin activity. This bidirectional relationship is particularly relevant in metabolic disorders, where dysbiosis contributes to disease progression. The review highlights recent findings on the roles of specific sirtuins in maintaining gut health and their implications in metabolic dysfunction and HCC development. Understanding these interactions offers potential therapeutic avenues for managing diseases linked to metabolic dysregulation and liver pathology.

## 1. Background

Sirtuins (SIRTs), classified as class III histone deacetylases (HDACs), are crucial regulators of various cellular mechanisms, particularly those related to ageing, metabolism, and disease [1,2]. These NAD^+^-dependent enzymes play a key role in maintaining cellular homeostasis by deacetylating histones, transcription factors, and other proteins, thereby influencing gene expression and metabolic pathways. Research increasingly highlights the significance of sirtuins in health conditions ranging from metabolic dysfunction to cancer, particularly hepatocellular carcinoma (HCC) [3].

The gut microbiota, a complex community of microorganisms residing in the intestinal tract, significantly impacts host health [4]. Emerging evidence indicates that sirtuins and gut microbiota significantly influence metabolic health and disease outcomes [4]. Sirtuins enhance gut barrier integrity and modulate immune responses, while gut microbiota-derived metabolites can affect sirtuin activity, creating a dynamic feedback loop that influences overall health [5]. Understanding the interplay between sirtuins and gut microbiota is essential for studying non-alcoholic fatty liver disease (NAFLD) and its progression to HCC [3]. As NAFLD becomes more prevalent, it is vital to uncover its underlying biological mechanisms to enable the development of effective treatments [6].

Previous studies have underscored the vital role of gut microbiota in the onset of metabolic diseases, particularly non-alcoholic fatty liver disease (NAFLD) and hepatocellular carcinoma (HCC). Dysbiosis in gut microbiota is linked to these metabolic conditions. Additionally, different studies also highlight the association of sirtuins with different metabolic diseases.

This review addresses how sirtuins influence gut microbial diversity and composition, as well as their relationship with metabolic diseases. We also explore how microbial metabolites affect sirtuin activity. Furthermore, we propose future research directions to investigate sirtuin-microbiota interactions, paving the way for new strategies to prevent and treat metabolic disorders and HCC. Integrating sirtuin and gut microbiota research represents a novel approach in this field, offering potential pathways for targeted therapeutic interventions.

## 2. Overview of Sirtuins and Their Biological Functions

### 2.1. Sirtuins Classification and Subcellular Localization

Sirtuins (SIRTs), a family of class III histone deacetylases (HDACs), play crucial roles in various cellular processes and have significant implications for ageing and related diseases [1,2]. They facilitate NAD^+^-dependent deacetylation processes that connect them with metabolic control. In terms of structure, sirtuins possess an N-terminal, a C-terminal, and a Zinc-binding domain. The seven types of mammalian sirtuins (SIRT1-7) feature a conserved catalytic core made up of 275 amino acids, consisting of two bi-lobed globular domains with NAD^+^ acting as a cofactor. The unique lengths, compositions, and post-translational modifications (PTM) of the N- and C-terminal domains differ, influencing their localization and functionality [7].

**Figure 1:**
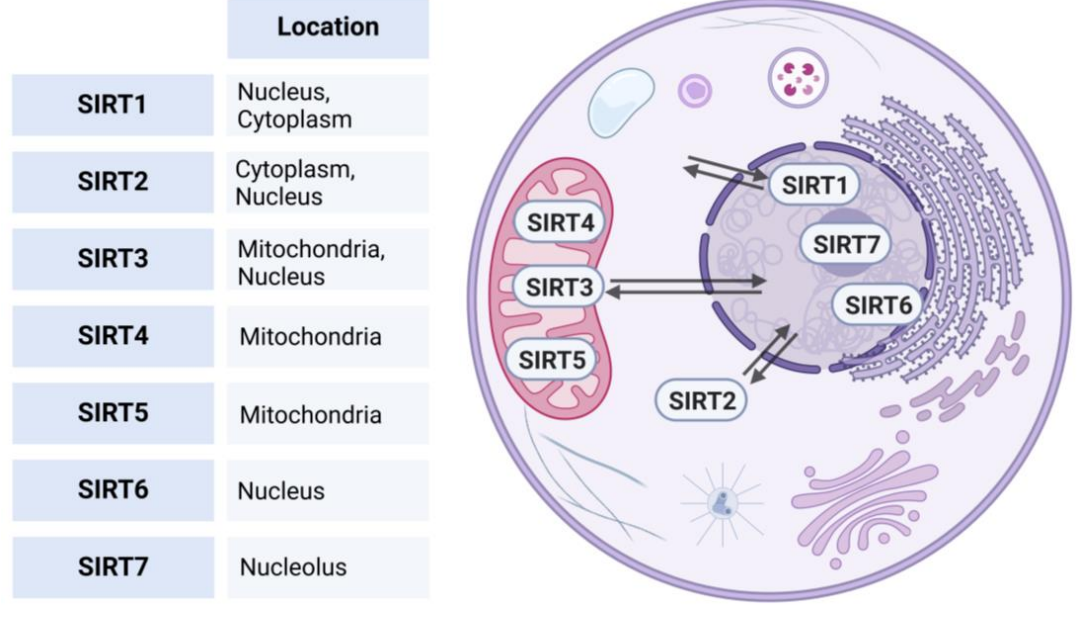
Subcellular localizations of human sirtuins. This figure illustrates the subcellular localization of human sirtuins (SIRT1-7). SIRT1, 6, and 7 predominantly reside in the nucleus; SIRT1 can translocate to the cytoplasm under physiological or pathological stimuli. SIRT2 is primarily located in the cytoplasm but can translocate to the nucleus. SIRT3 is located in the mitochondria and is able to relocate between the mitochondria and nucleus, while SIRT4 and SIRT5 are typically found in the mitochondrial compartment. Created in BioRender. https://BioRender.com/r59u349 (accessed on 12 March 2025).

Phylogenetic analysis based on the amino acid sequence revealed four different classes of sirtuins. SIRT1-3 belong to Class I, SIRT-4 to Class II, SIRT 5 to Class III, SIRT6-7 to Class IV [7]. Each protein exhibits distinct subcellular compartmentalization [8]. SIRT1, 6, and 7 predominantly reside within the nucleus. However, SIRT1 can translocate to the cytoplasm under physiological or pathological stimuli [9]. SIRT2, on the other hand, is primarily situated within the cytoplasm but is capable of translocating to the nucleus under certain conditions to influence cellular processes [10]. Additionally, SIRT3 can also undergo relocation between the mitochondria and the nucleus, enabling it, to regulate a wide range of cellular processes [11], while SIRT 4, and 5 are typically located in the mitochondrial compartment (see Figure 1) [7,12].

These dynamic subcellular distributions and trafficking abilities allow the sirtuins to coordinate their regulatory functions across different compartments of the cell [13]. SIRTs play a key regulatory role in metabolism, health-span, and longevity by deacetylating histones, transcriptional regulators, and other proteins, in addition to their ADP-ribosyltransferase and deacetylase activities [2,14].

### 2.2. Role of Sirtuins in Cellular Homeostasis and Metabolism

SIRTs are involved in post-translational modifications, particularly protein deacetylation, and contribute to gene expression regulation, DNA repair, and cellular senescence [15,16]. These enzymes enhance genomic stability through chromatin structural modulation and involvement in various DNA repair mechanisms, including base excision repair, nucleotide excision repair, and double-strand break repair [17,18]. SIRTs also serve a crucial role in the modification of histones and proteins, influencing cellular metabolism, mitochondrial function, and stem cell maintenance [19,20]. SIRT1, the most widely studied sirtuin, is recognised for its role in transcriptional regulation, genomic silencing, and epigenetic elements within the nucleus, while also contributing to metabolism and nutrient perception in the cytosol [21]. The involvement of SIRTs in ageing and cancer has been extensively studied, with different sirtuin family members showing diverse effects on stem cells and cancer cells [22,23,24].

SIRTs play vital roles in various physiological and pathological conditions, such as neurodegenerative diseases [25], kidney disorders [26], and cardiovascular diseases [8,27]. They are important for maintaining cellular integrity [28] and regulating metabolic balance, including glucose and lipid metabolism [29]. Additionally, they modulate mitochondrial activity and have been associated with various metabolic disorders [8]. SIRTs also contribute to the regulation of female reproductive processes [30] and influence women’s health, particularly in ovarian function and cancer development [31]. In the immune system, SIRTs modulate T cell metabolism and function, making them promising therapeutic targets for immune-related diseases [32]. Furthermore, SIRTs have been implicated in counteracting several hallmarks of ageing, potentially contributing to healthy longevity [33].

Different investigations underscore the crucial and multifaceted functions of sirtuins in neoplastic advancement, acting as tumour suppressors as well as activators of tumourigenesis, depending on the specific cellular environment [34,35]. SIRTs regulate various cancer-related processes, including cell viability, apoptosis, metastasis, and metabolism [34,36]. Among all SIRTs, SIRT1 has been extensively researched for its dual role in cancer, contributing to tumour suppression and promotion [21,37]. SIRT2′s role remains controversial, with evidence suggesting it can act as either an oncogene or a tumour suppressor across multiple malignancies [38]. The roles of other sirtuins (SIRT3-7) vary across different cancer types, influencing processes such as proliferation, invasion, and chemoresistance [39,40,41]. Furthermore, SIRTs play crucial roles in hepatocellular carcinoma (HCC) development and progression [3,42]. SIRT1 is overexpressed in HCC, promoting oncogenesis and multidrug resistance [43]. Collectively, these findings emphasise sirtuins’ vital role in cellular homeostasis and disease management, particularly in cancer, necessitating further investigation into their specific functions and therapeutic implications. (see Table 1).

## 3. The Role of Gut Microbiota in Host Health and Disease

The gut microbiota includes a variety of microorganisms, such as bacteria, archaea, fungi, viruses, and parasites [69,70,71]. These microorganisms, inhabit within the gastrointestinal tract—particularly in the intestinal—and are commonly designated as gut flora or gut microbiota [72]. While bacteria dominate the gut microbiome, the significance of other microbes—often referred to as the “dark matter” of microbiomes—has become increasingly recognised [70]. The predominant bacterial phyla present within the human gut microbiome include Firmicutes, Bacteroidetes, Proteobacteria, Actinobacteria, and Fusobacteria [73]. Furthermore, this complex community plays essential roles in host physiology, metabolism, and immune function [74,75]. The gut microbiota helps maintain intestinal integrity through the production of microbial metabolites and anti-microbial peptides modulating the immunity [76]. Additionally, it serves as a potential source of novel antimicrobials, which could help address antimicrobial resistance [77]. Moreover, the gut microbiota contributes significantly to digestion by breaking down indigestible dietary components and producing essential nutrients like vitamins and enzymes [78]. Furthermore, the microbiota plays a critical role in modulating inflammation, cancer-related processes, and oxidative stress through the production of metabolites, especially short-chain fatty acids (SCFAs) and tryptophan catabolites [79,80,81,82].

Gut microbiota are essential for the fermentation of non-digestible carbohydrates, yielding SCFAs such as acetate, propionate, and butyrate [83,84]. These SCFAs serve as energy sources for colonocytes and maintain gut barrier integrity [85,86]. Moreover, SCFAs influence host metabolic processes and immune responses via diverse mechanisms, such as the stimulation of G-protein-coupled receptors and the suppression of histone deacetylases [87]. The gut microbiota is also participates in bile acid metabolism, which is essential for lipid digestion and absorption [88]. Consequently, microbial SCFA production and bile acid metabolism significantly impact host health via intricate interactions with the gut epithelium, immune response, and metabolic pathways [78,89]. Grasping these mechanisms is essential for developing approaches to enhance gut health and avoid metabolic diseases.

**Figure 2:**
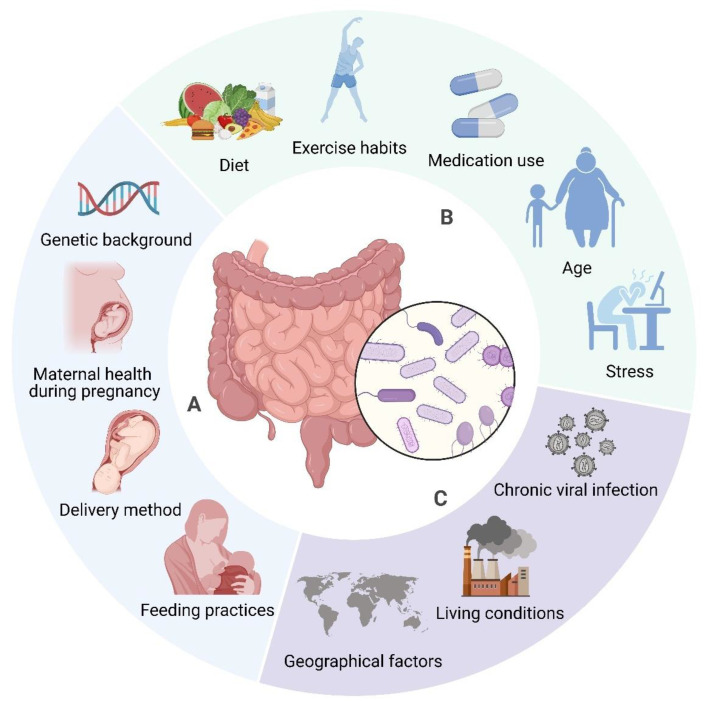
Determinants of Gut Microbiome Composition. The composition of gut microbiota is affected by a wide range of factors. Some elements, such as genetic background (e.g., specific gene variants), delivery method (vaginal vs. caesarean), early infant feeding practises (breastfeeding vs. formula feeding), and maternal health during pregnancy (e.g., gestational diabetes, infections) are established early in life and tend to remain stable (A). In contrast, factors like diet (e.g., high fibre vs. high fat), medication use (antibiotics), exercise habits, age, and stress levels are more variable and can be modified throughout life (B). Furthermore, environmental influences, including chronic viral infections (e.g., Epstein–Barr virus), living conditions (urban vs. rural), and geographical factors (climate, culture), also contribute to microbiome composition, although these may be more challenging to alter (C). Created in BioRender. https://BioRender.com/r59u349 (accessed on 12 March 2025).

The composition of gut microbiota is modulated by factors including diet, genetics, and environmental conditions [90,91,92]. Diet significantly influences the microbiome, alongside seasonal and geographical factors impacting dietary habits and microbial composition [93]. Environmental determinants, such as communal residences, exert a more significant influence on microbiome composition than host genetics [94]. Nevertheless, host genetics continue to exert an influence in ascertaining microbiome composition, especially via immune system-related genes [95]. Early life factors, including birth mode, feeding methods, and antibiotic use, also contribute to microbiota development [96]. The gut microbiome is dynamic, changing throughout an individual’s lifetime due to factors like age, BMI, exercise, and lifestyle (see Figure 2) [92]. Recognising these influences is important for maintaining a healthy microbiome and preventing dysbiosis, which has been linked to several diseases [91,97].

Dysbiosis, characterised by a perturbation in the assemblage of gastrointestinal microbiota, has been associated with numerous pathological states, encompassing inflammatory bowel disease (IBD), cardiovascular diseases, diabetes mellitus, obesity, and various neurological disorders [98,99,100,101,102]. This dysbiosis frequently presents as a diminution in microbial variety alongside an elevation in particular bacterial taxa [101]. Factors contributing to dysbiosis include antibiotic use, diet, and environmental stressors [100,101]. Dysbiosis can lead to altered microbial metabolite production, immune dysregulation, and chronic inflammation [103]. Therapeutic modalities aimed at addressing dysbiosis encompass faecal microbiota transplantation, the administration of probiotics, the utilisation of prebiotics, as well as various dietary interventions [104,105].

## 4. Interrelationship Between Sirtuins and Gut Microbiota: A Bidirectional Perspective

### 4.1. Influence of Sirtuins on Gut Microbiota Composition

Sirtuins are essential in gut function regulation, particularly in preserving the intestinal barrier and mucosal immune mechanism, which are vital for regulating intestinal microbiota composition [106].

#### 4.1.1. SIRT1

Intestinal epithelial SIRT1 regulates gut microbial composition consequently preventing age-related intestinal inflammation [107]. The gut microbiome remotely regulates the expression of miR-204 subsequently impairing the endothelial function by targeting Sirt1 [108]. SIRT1 deficiency in the intestinal epithelium leads to increased faecal bile acid levels, reduced *Lactobacillus* abundance, and heightened susceptibility to intestinal inflammation and colitis [109]. Moreover, the gut microbiome modulates systemic hydrogen sulphide levels impacting SIRT1 activation followed by the regulation of neuroinflammation [110].

#### 4.1.2. SIRT2

Similarly, SIRT2 deficiency enhances the progression of NAFLD by altering gut microbiota composition and inducing metabolic disorders [6], whereas inhibiting SIRT2 may improve gut barrier integrity and protect against colitis [111]. SIRT2 knockout mouse displayed increased susceptibility to obesity, liver injury, and metabolic dysfunction when placed on high fat/high sucrose/high cholesterol diet [6].

#### 4.1.3. SIRT3

SIRT3 and the gut microbiota interaction leads to modulation of key cellular processes like mitochondrial function, inflammation, and energy metabolism [112]. SIRT3 deficiency promotes NAFLD progression through gut microbial dysbiosis and impaired intestinal permeability [113]. Moreover, the gut microbiota modulates hydrogen sulphide levels consequently affecting SIRT3 activation [110].

#### 4.1.4. SIRT4

Mitochondrial SIRT4 regulates intestinal metabolism and homeostasis. It regulates lysozyme expression in the gut, influencing microbiota composition [114]. Loss of SIRT4 can lead to dysregulated glutamine and nucleotide metabolism in intestinal adenomas [115].

#### 4.1.5. SIRT5-7

While the roles of SIRT5, SIRT6, and SIRT7 in gut microbiota regulation are not directly established, it is notable that downregulation of SIRT5 has been linked to increased bile acid production, which may contribute to an immunosuppressive tumour microenvironment and facilitate hepatocellular carcinoma (HCC) development [116]. Moreover, SIRT6 knockout mice exhibit premature ageing associated with gut dysbiosis, a condition reversible through faecal microbiota transplantation or a high-fat diet [117]. It is noteworthy that the presence of an inflammatory response of the colorectal mucosa is associated with higher concentrations of SIRT7 and lower concentrations of SIRT1 [118].

Together, these roles underscore the importance of sirtuins in gut health and their potential influence on microbiota dynamics, highlighting areas for future research (see Table 2).

### 4.2. Impact of Gut Microbiota on Sirtuin Activity

Conversely, gut microbiota also influences sirtuin activity and expression. Studies indicate that gut microbiota can regulate the expression of sirtuins, along with senescence-regulating miRNAs and mitochondrial DNA, all associated with overall well-being [131]. Moreover, gut microbiota interacts with sirtuin-activating compounds to influence molecular pathways that counteract ageing and inflammation, while enhancing specific gut microbiota groups to improve immune function [132].

Studies have elucidated that gastrointestinal microbiota may influence critical transcriptional co-activators, transcription factors, and enzymatic pathways pertinent to mitochondrial biogenesis, encompassing genes such as PGC-1α, SIRT1, and AMPK [133]. Additionally, the gut microbiome regulates vascular microRNA-204, which targets SIRT1 and affects endothelial function [108]. Furthermore, metabolites produced by gut microbiota from fermenting indigestible food components can impact sirtuin function [134]. For instance, gut microbiota-derived metabolites have anti-inflammatory and antioxidative properties that influence sirtuin activity [135]. Particularly, Urolithin A (UroA), a microbial metabolite derived from polyphenolics, exemplifies this by exhibiting anti-inflammatory and antioxidative effects [135]. These properties of microbial metabolites can directly affect sirtuin function, as sirtuins are involved in regulating oxidative stress and inflammation [136]. By modulating these pathways, gut microbiota-derived metabolites can indirectly affect sirtuin activity and contribute to overall host health.

Additionally, the gut microbiome possesses the capacity to affect sirtuin-associated pathways; for example, *Saccharomyces boulardii* has been demonstrated to alter necrotizing enterocolitis through the modulation of the SIRT1/NF-κB signalling pathway and the intestinal microbiota [137]. The overall outcomes shed light on the intricate relationship between sirtuins and gut microbiota, stressing the requirement for more extensive investigation to clarify current shortcomings in understanding their roles and interconnections.

## 5. Role of Sirtuins and Gut Microbiota in Non-Alcoholic Fatty Liver Disease (NAFLD)

NAFLD represents the most widespread form of chronic hepatic disorder globally and is projected to emerge as the primary etiology for liver transplantation by the year 2030 [138]. Furthermore, NAFLD is distinguished by an atypical aggregation of hepatic lipids, concomitant with insulin resistance, and demonstrable steatosis, while systematically ruling out secondary etiologies of hepatic steatosis, such as alcohol consumption [139]. Additionally, NAFLD comprises a spectrum of disorders extending from hepatic steatosis to steatohepatitis, leading to inflammation, hepatocirrhosis, hepatocellular carcinoma, and mortality [140]. Also, it is closely associated with insulin resistance and may function as both a cause and a result of metabolic syndrome [141].

Formerly, the “two-hit” hypothesis previously explained NAFLD pathogenesis, identifying lipid accumulation as the “first hit”. This initial accumulation of lipid predisposes the liver to further injury, termed the “second hit”, resulting in inflammation and fibrosis [142]. Recent research proposes the multiple-hit hypothesis, which more comprehensively describes the molecular and metabolic alterations in NAFLD. This updated framework includes interconnected mechanisms, such as insulin resistance, lipotoxicity, innate immune activation, and gut microbiome effects, influenced by genetic (PNPLA3) and dietary elements (saturated fat and fructose) [142].

The interplay between sirtuins and intestinal microbiota is a complex relationship. The gut microbiota plays a crucial role in host metabolism by breaking down nutrients and producing metabolites that influence metabolic processes and modulate immunity [143]. Sirtuins are recognised for their ability to govern the intestinal microbiome, thereby suggesting their participation in the pathophysiological mechanisms underlying a variety of diseases. They are integral to numerous physiological processes, encompassing glucose and lipid metabolism, insulin resistance, and mitochondrial function, thereby rendering them pivotal factors in the etiology of conditions such as type 2 diabetes, obesity, and NAFLD [107,144,145].

Furthermore, sirtuins are influenced essential metabolic hormones such as leptin, ghrelin, melatonin, insulin, and serotonin, which perform a crucial function in the regulation of gastrointestinal homeostasis [146]. Ghrelin, leptin, and insulin also play a critical role in neural plasticity and cognition [147]. SIRT1 has been documented to be associated with metabolic homeostasis melatonin activates SIRT1 in order to combat mitochondrial dysfunction, inflammation, and oxidative stress [148,149]. Ghrelin and ghrelin potentiator treatment can not only increase SIRT1 expression at the protein level but also its activity [150]. Leptin promotes the expression of SIRT1 by activating Nrf2, which may play a role in colon cancer development [151]. Additionally, leptin reduces the expression of BACE1 and the production of amyloid-β by triggering the SIRT1 signalling pathway, thereby diminishing NF-κB-driven transcription of BACE1 [152]. There is a connection between age-related weight gain and leptin resistance due to lower levels of SIRT1 in the hypothalamus. Preserving SIRT1 activity in the hypothalamus could enhance leptin sensitivity and help counteract weight gain associated with ageing [153]. Moreover, the gut bacteria also tend to influence the level of peptides like leptin, ghrelin, and melatonin [154].

Recent studies indicate a significant reduction in sirtuin levels in NAFLD patients. Wu et al. found decreased expression of SIRT1, SIRT3, SIRT5, and SIRT6 in NAFLD patients, alongside increased lipogenic gene expression and SIRT4 [155]. Furthermore, Bruce et al. demonstrated that excess dietary fat exposure during early and postnatal periods elevates NASH risk in adulthood, particularly affecting sirtuin levels. Offspring on a high-fat diet exhibited NAFLD, with those from high-fat diet mothers developing NASH, characterised by decreased NAD^+^/NADH and lower SIRT1 and SIRT3 levels, coupled with increased lipid metabolism gene expression [156].

**Figure 3:**
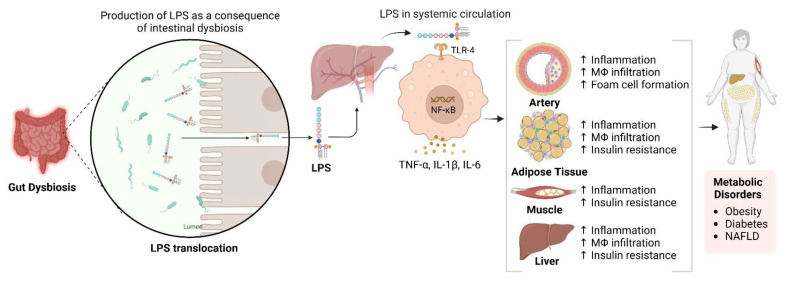
Impact of Gut Dysbiosis on Inflammation and Metabolic Dysfunction. This figure illustrates the process following gut dysbiosis, which leads to the release of lipopolysaccharides (LPS). These LPS molecules enter the systemic circulation via the portal vein, activating macrophages through Toll-like receptor 4 (TLR4). This activation results in the release of pro-inflammatory cytokines, including TNF-α, IL-1β, and IL-6, contributing to increased inflammation and hepatic insulin resistance. Additionally, activated macrophages infiltrate adipose tissue, exacerbating inflammation and insulin resistance, thereby contributing to metabolic disorders such as obesity, diabetes, and non-alcoholic fatty liver disease (NAFLD). The diagram also indicates the systemic effects of inflammation on various tissues, including the arteries, adipose tissue, muscle, and liver. Created in BioRender. https://BioRender.com/r59u349 (accessed on 12 March 2025).

The gut microbiota is essential for NAFLD development through dietary metabolism, yielding vital nutrients and energy [157,158,159]. Its composition is diet-dependent, with high-fat/high-cholesterol (HFC) and high-fat/high-sucrose (HFS) diets causing dysbiosis linked to NAFLD [160,161,162]. This dysbiosis increases intestinal permeability, exposing the liver to bacterial products through the portal vein and inducing metabolic endotoxemia, thereby disrupting the gut-liver axis [163,164]. NAFLD microbiota differs from that of healthy individuals and is influenced by genetic factors related to metabolic syndrome 170]. Additionally, gastrointestinal microbiomes can synthesise lipopolysaccharides (LPS), which may infiltrate the circulatory system and impair the hepatic tissue when the intestinal barrier is compromised (see Figure 3) 171]. In contrast, SCFAs such as butyrate are crucial for maintaining gut barrier integrity. Zhou et al. demonstrated that sodium butyrate enhances gut microbiota and fortifies the intestinal barrier, mitigating LPS translocation and reducing steatohepatitis in mice [165]. This underscores the significance of a robust gut barrier.

Chen et al. demonstrated that SIRT3 deficiency worsens NAFLD from a high-fat diet through impaired intestinal permeability linked to gut microbial dysbiosis in SIRT3 knockout mice. The SIRT3KO mice exhibited increased Oscillibacter, Parabacteroides, and Mucispirillum, while Alloprevotella decreased [113]. Prior studies have shown a negative correlation between Oscillibacter levels and the mRNA expression of zonula occludens-1, a protein relevant to gut permeability [166,167,168]. Furthermore, Everard et al. noted increased Mucispirillum and Parabacteroides in humans and mice on a high-fat diet [169]. The elevation of Oscillibacter, Mucispirillum, and Parabacteroides correlates with exacerbated HFD-induced NAFLD in SIRT3KO mice, characterised by diminished tight junction protein expression like ZO-1 and claudins [113]. Thus, SIRT3-mediated intestinal barrier dysfunction, coupled with LPS release from gut microbiome alterations, facilitates NAFLD progression.

## 6. Role of Sirtuins and Gut Microbiota in Hepatocellular Carcinoma (HCC)

Liver cancer represents a considerable global health issue, with rising rates primarily attributed to hepatocellular carcinoma (HCC), which constitutes about 90% of cases [170,171]. Significant risk factors for HCC development include chronic hepatitis B and C virus infections. Non-viral factors involve environmental carcinogens such as aflatoxin B1, alcohol misuse, and genetic conditions like hemochromatosis and Wilson disease [172]. Importantly, non-alcoholic steatohepatitis (NASH) has emerged as a critical criterion for liver transplantation among HCC patients in the United States [173]. The transition from NAFLD to HCC entails a multifaceted interaction of metabolic variables, inflammation, gut microbiota imbalance, oxidative stress, and aberrant lipid metabolism [174,175,176]. Dysbiosis, has been implicated in the pathogenesis of HCC and its related chronic diseases, which encompass chronic hepatitis B and C, alcoholic liver disease, NAFLD, and NASH [177].

Sirtuins play a complex role in cancer development, including HCC. Their dual nature in cancer biology is evident, as they can function as both promoters and suppressors of tumours, depending on the cellular context and the specific sirtuin involved. This complexity is particularly significant in relation to DNA repair, genomic stability, cell cycle regulation, apoptosis, metabolism, and the oxidative stress response [34,178].

SIRT1 has a critical role in HCC by virtue of promoting tumourigenicity, metastasis, chemoresistance, and heralding a poor prognosis [43,179,180]. The interplay between SIRT1 and intestinal microbiota is complex. Butyrate, a short-chain fatty acid from gut bacteria, is crucial in this relationship. A decrease in butyrate-producing bacteria may harm the intestinal mucosa, possibly leading to HCC development [181]. Pant et al. illustrated that butyrate induces microRNA-22 (miR-22), which downregulates SIRT1 in a concentration-dependent manner, increasing reactive oxygen species (ROS) and apoptosis in hepatic cells exposed to sodium butyrate [182]. MiR-22′s downregulation in human liver cancer cells correlates with enhanced tumourigenicity and cellular proliferation [183]. It is implicated in HCC development via the miR-29a-SIRT1-WNT/β-catenin pathway [184]. Furthermore, overexpression of SIRT1 promotes HCC proliferation and resistance to chemotherapy by promoting autophagy [185,186]. Moreover, SIRT1 protects mitochondrial function of HCC cells by suppressing the expression of hypoxia-induced factor-1 alpha expression and also promotes stem-cell like features in HCC cells [187].

SIRT1 also promotes HCC metastasis by enhancing PGC-1α-mediated mitochondrial biogenesis [188]. Conversely, Herranz et al. reported that SIRT1 overexpression can protect transgenic animals from diethylnitrosamine/high-fat diet-induced liver cancer by mitigating NF-kB-mediated inflammation and averting malignant transformation [189]. This observation contrasts with earlier assertions of SIRT1′s role in promoting HCC and other studies indicating elevated SIRT1 in human HCC samples [190,191]. Malignant cells may utilise survival strategies typically reserved for non-malignant cells [192]. Portmann et al. have demonstrated that inhibiting SIRT1 leads to impaired tumour growth both in vivo and in vitro and this supports the notion that SIRT1 activity in healthy hepatocytes protects against cancer, but after transformation, SIRT1 becomes a protective force for the tumour cells as a survival advantage [193]. However, additional research is essential to elucidate the intricate relationship between butyrate and SIRT1 in the etiology of HCC.

SIRT2 mediates the deacetylation and activation of protein kinase B, impacting the glycogen synthase kinase-3β/β-catenin signalling pathway, which is implicated in epithelial–mesenchymal transition (EMT) [194]. Huang et al. support the tumour-promoting role of SIRT2, revealing that its downregulation hinders energy metabolism and invasion in HCC cells [195]. Further investigation is warranted to elucidate SIRT2′s specific functions in HCC.

SIRT3 plays the role of a tumour suppressor in HCC. SIRT3 is downregulated in HCC tumour tissue compared to adjacent non-cancerous tissues [196,197]. Overexpression of SIRT3 decreases HCC cell proliferation and promotes apoptosis through multiple mechanisms, including the activation of the GSK-3β/Bax signalling pathway [196], upregulation of p53 [198], and reduction in Mdm2-mediated p53 degradation [199]. SIRT3 also decreases GSTP1 levels and activates the JNK signalling pathway [200], which further increases the efficacy of chemotherapeutic agents on HCC cells.

SIRT4 and SIRT5 also play significant roles in HCC. SIRT4 depletion promotes HCC tumour development through the AMPKα/mTOR pathway [201], while overexpression of SIRT4 induces G2/M cell cycle arrest and apoptosis, leading to tumour suppression [202]. SIRT5, on the other hand, is associated with the progression of HCC through its effects on mitochondrial apoptosis pathways [203]. It also promotes HCC cell proliferation and invasion by targeting E2F1 [204].

SIRT6 plays a complex role in HCC. While some studies suggest SIRT6 acts as a tumour promoter by preventing DNA damage and cellular senescence [205], others indicate that it suppresses tumour growth by inhibiting the ERK1/2 pathway [206]. SIRT6 overexpression has been associated with increased HCC cell proliferation and apoptosis evasion [207,208]. However, SIRT6 deficiency in knockout (HKO) mice enhances liver tumourigenesis via ERK1/2 pathway activation. Overexpression of SIRT6, particularly in the HuH7 liver cancer cell line and xenograft mouse model, inhibits tumour growth, suggesting its potential as a therapeutic target for HCC [209]. The contradictory findings highlight the need for further research to clarify SIRT6′s role in HCC and its potential as a therapeutic target [3].

SIRT7 plays a multifaceted role in HCC, with its upregulation noted in numerous patients [210]. It facilitates HCC cell proliferation through ERK1/2 phosphorylation and activation of the RAF/MEK/ERK signalling cascade, promoting tumour growth [211]. Moreover, SIRT7 boosts HCC cell proliferation by inhibiting MST1 and modulating the Hippo/YAP pathway, resulting in enhanced YAP activation [212].

In summary, the interplay between sirtuins and gut microbiota is essential for elucidating the pathogenesis of NAFLD and HCC. Table 3 highlights the microbial changes linked to these diseases, indicating opportunities for specific therapeutic interventions.

Therapeutic approaches aimed at the gut microbiome, including prebiotics, probiotics, and faecal microbiota transplant, appear to hold potential for addressing metabolic conditions and age-related diseases [213]. However, challenges remain in translating research findings into effective clinical applications due to the complex nature of host-microbiota interactions and methodological concerns [214]. Additional research is required to clarify specific mechanisms and to create targeted treatments for different health issues. Subsequent investigations should aim to utilise these findings to formulate of microbiota-targeted therapies to improve disease management and patient prognosis.

**Table 3:** Microbiota composition changes in NAFLD and HCC: A summary of human studies.

Disease	Composition Change		References
	Increase	Decrease	
**NAFLD**	*Streptococcus*, *Megasphaera*, *Enterobacteriaceae*, *Streptococcus*, *Gallibacterium*	*Bacillus* and *Lactococcus*, *Pseudomonas*, *Faecalibacterium prausnitzii*, *Catenibacterium*, *Rikenellaceae*, *Mogibacterium*, *Peptostreptococcaceae*	[215]
	*Firmicutes* (*Streptococcus mitis* and *Roseburia inulinivorans*) and *Bacteroidetes* (*Barnesiella intestinihominis* and *Bacteroides uniformis*)	*Bacteroidetes* (*Prevotella* sp. CAG 520, *Prevotella* sp. AM42 24, *Butyricimonas virosa*, and *Odoribacter splanchnicus*), *Proteobacteria* (*Escherichia coli*), *Lentisphaerae* (*Victivallis vadensis*), and *Firmicutes* (*Holdemanella biformis*, *Dorea longicatena*, *Allisonella histaminiformans*, and *Blautia obeum*)	[216]
	*Bacteroidetes*, *Proteobacteria*, *Bacteroides*, *Alistipes*, *Verrucomicrobia*, *Faecalibaculum*, *Helicobacter*, *Epsilonbacteraeota*	*Muribaculaceae*, *Lactobacillus*	[217]
**HCC**	*Escherichia coli*		[218]
	*Proteobacteria*, *Desulfococcus*, *Enterobacter*, *Prevotella*, *Veillonella*	*Cetobacterium*	[219]
	*Bacteroides*	*Akkermansia*, *Bifidobacterium*	[220]
	*Neisseria*, *Enterobacteriaceae*, *Veillonella*, *Limnobacter*	*Enterococcus*, *Phyllobacterium*, *lostridium*, *Ruminococcus*, *Coprococcus*	[221]
	*Proteobacteria*, *Enterobacteriaceae*, *Bacteroides xylanisolvens*, *B. caecimuris*, *Ruminococcus gnavus*, *Clostridium bolteae*, *Veillonella parvula*	*Erysipelotrichaceae*, *Oscillospiraceae*	[222]
	*Klebsiella*, *Haemophilus*	*Alistipes*, *Phascolarctobacterium*, *Ruminococcus*	[181]

## 7. Interventions Targeting Sirtuins and Gut Microbiota

SIRTs are pivotal in linking health and disease, highlighting their potential for targeting interventions [223]. The healthcare sector increasingly recognises the therapeutic value of manipulating the sirtuin pathway and gut microbiome in disease management [146]. SIRT1 and SIRT2 have emerged as promising targets for therapeutic interventions in various diseases associated with gut dysbiosis, including cancer, neurodegenerative disorders, and metabolic conditions [6,146,224]. However, studies demonstrating the pharmacological utility of modifying other sirtuins in diseases where gut dysbiosis contributes to the underlying mechanisms remain limited.

Current strategies for modulating sirtuin activity in the treatment of hepatocellular carcinoma (HCC) and non-alcoholic fatty liver disease (NAFLD) focus on the activation and inhibition of specific sirtuins, particularly SIRT1 and SIRT2, respectively. These approaches aim to exploit the complex roles of sirtuins in HCC progression, chemoresistance, and the underlying mechanisms of NAFLD. Various strategies, including pharmacological agents, gene editing, and small molecule inhibitors, are being explored to enhance or inhibit sirtuin functions, making this modulation a promising avenue for cancer therapy [3,225].

### 7.1. Sirtuin Activators

#### 7.1.1. Resveratrol

**Figure 4:**
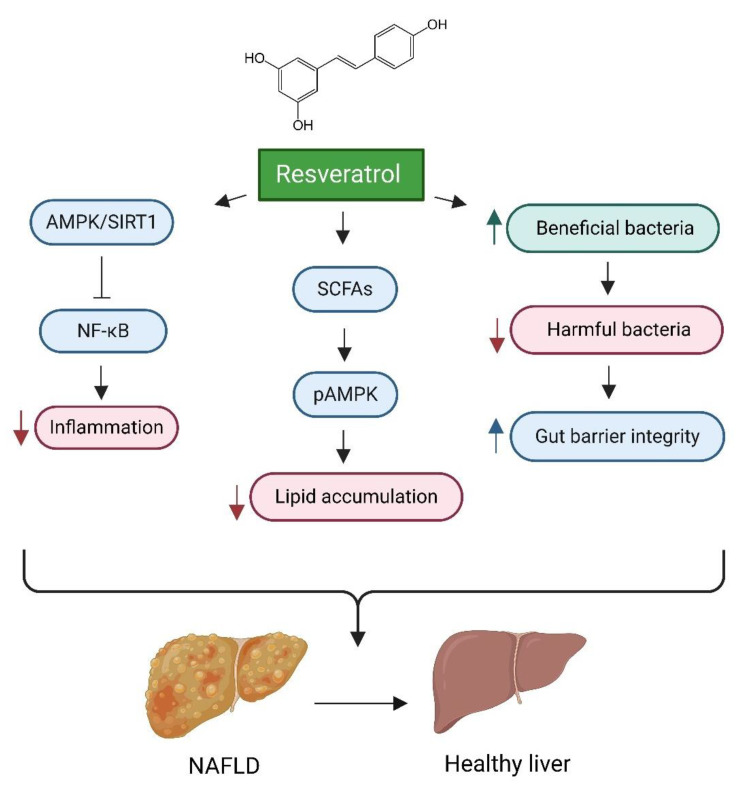
Impact of Resveratrol on Gut Health and Liver Disease. This figure illustrates the multifaceted mechanisms through which resveratrol mitigates the progression of non-alcoholic fatty liver disease (NAFLD) and improves liver health. Key processes include the enhancement of gut microbiota by promoting beneficial bacterial proliferation, which strengthens gut barrier integrity and overall gut health. Additionally, resveratrol activates the AMPKα/SIRT1 signalling pathway, suppressing the NF-κB inflammatory pathway and leading to decreased inflammation and hepatic steatosis. Furthermore, resveratrol influences hepatic lipid metabolism and reduces lipid accumulation by increasing the production of short-chain fatty acids (SCFAs), which enhances AMPK activation and contributes to decreased lipid accumulation. Created in BioRender. https://BioRender.com/r59u349 (accessed on 12 March 2025).

One prominent example of therapeutic intervention is the targeted activation of the SIRT1 pathway [226]. Resveratrol, a polyphenolic compound, has demonstrated efficacy in mitigating the progression of NAFLD in both preclinical and clinical contexts [227]. Resveratrol enhances gut microbiota by promoting the proliferation of beneficial bacteria, thereby improving gut health. This effect is characterised by a decrease in harmful bacteria and an increase in short-chain fatty acid (SCFA)-producing bacteria, which contributes to overall gut microbiota balance and metabolic health [228]. Furthermore, resveratrol alleviates NAFLD by strengthening gut barrier integrity, reducing inflammation, and increasing the production of short-chain fatty acids (SCFAs) [229,230]. Additionally, it exhibits potential protective effects against HCC through the modulation of inflammatory, angiogenic, and oxidative stress pathways [231]. Resveratrol’s proposed mechanism of action involves the modulation of hepatic lipid metabolism and the reduction in oxidative stress, primarily through the activation of the AMPKα/SIRT1 signalling pathway. This activation effectively suppresses the nuclear factor kappa B (NF-κB) inflammatory pathway, resulting in decreased inflammation and reduced hepatic steatosis [232,233]. (see Figure 4).

#### 7.1.2. Pterostilbene

Pterostilbene, a dimethyl ether variant of resveratrol, exhibits encouraging effects against NAFLD and obesity. It diminishes liver fat accumulation by influencing the miR-34a/Sirt1/SREBP-1 pathway in rats fed a fructose diet [234]. Compared to resveratrol, pterostilbene has enhanced bioavailability and metabolic stability [235]. In rats consuming a high-calorie diet, pterostilbene lowers adipose tissue volume, inhibits lipogenesis in fat tissue, and promotes fatty acid oxidation in the liver [236]. From a metabolic perspective, pterostilbene displays greater stability than resveratrol and often shows more potent pharmacological effects [237]. Taken together, these studies indicate that pterostilbene is a promising candidate for addressing NAFLD and obesity, with potential benefits over resveratrol due to its superior bioavailability and metabolic characteristics.

#### 7.1.3. E1231

E1231, treatment activates SIRT1 alleviating NAFLD by regulating lipid metabolism. Moreover, E1231 prevented lipid accumulation and improved mitochondrial function in free fatty acid challenged hepatocytes. E1231 prevented liver injury via regulation of SIRT1 and AMPK-α pathway [238].

#### 7.1.4. Quercetin

Quercetin, a natural flavonoid present in plants, has demonstrated encouraging effects in the treatment of NAFLD. Laboratory studies indicate that quercetin diminishes lipid buildup, lowers inflammatory cytokines, and boosts antioxidant activity in liver cells [239]. Animal studies show that quercetin helps improve NAFLD by triggering AMPK-mediated mitophagy, reducing lipid storage, and alleviating oxidative stress [240]. Supplementing the diet with quercetin in gerbils suffering from high-fat diet-induced NASH led to better lipid profiles, a decline in inflammatory markers, and regulation of Sirt1 and NF-κB p65 expression [241]. Thus, the hepatoprotective properties of quercetin are linked to enhanced fatty acid metabolism, anti-inflammatory and antioxidant effects, and modulation of gut microbiota as well as bile acids [242]. These results emphasise the potential of quercetin as a therapeutic option for NAFLD.

#### 7.1.5. Nicotinamide Riboside (NR)

Nicotinamide riboside (NR), an NAD^+^ precursor, shows promise in addressing NAFLD and its progression to HCC. NR supplementation reduces hepatic lipid accumulation, inflammation, and fibrosis in various NAFLD models [243,244,245]. It activates SIRT1/AMPK-mediated browning of white adipose tissue and modulates gut microbiota, potentially improving lipid metabolism [246]. Furthermore, NR supplementation alleviated weight loss, reduced metastasis, and extended survival in BALB/c nude (xenograft) and C57BL/6J (allograft) mouse models with HCC by boosting NAD^+^ levels [247].

#### 7.1.6. Berberine

Berberine, a natural plant alkaloid, has emerged as a promising therapeutic agent for NAFLD and its progression to HCC [248]. It exerts beneficial effects on gut microbiota by promoting the growth of beneficial bacterial populations while reducing pathogenic strains [249,250]. Additionally, berberine enhances lipid metabolism, improves insulin sensitivity, and diminishes inflammation [248,251]. Berberine has beneficial effects on NAFLD through various molecular pathways, including the activation of SIRT3, SIRT1, AMPK, and PPAR-γ, as well as the suppression of the NLRP3 pathway [251]. Additionally, berberine activates intestinal farnesoid X receptor (FXR), resulting in increased expression of fibroblast growth factor 15 (FGF15), which in turn inhibits lipogenesis and the activation of NF-κB pathway in the liver [250]. Furthermore, berberine suppresses the p38 MAPK/ERK-COX2 signalling pathways, thereby reducing inflammation and angiogenesis associated with non-alcoholic steatohepatitis (NASH) and HCC [252].

#### 7.1.7. Yinchen Linggui Zhugan Decoction (YLZD)

Yinchen Linggui Zhugan decoction (YLZD), a traditional Chinese medicine, has demonstrated promise in treating NAFLD by modulating the SIRT1/Nrf2 pathway and gut microbiota. In Sprague Dawley (SD) rat models, YLZD treatment has been shown to reduce NAFLD induced by a high-fat diet, increasing serum and faecal butyric acid levels and total short-chain fatty acids (SCFAs), while promoting a favourable shift in gut microbiota composition towards SCFA-producing bacteria [253].

#### 7.1.8. The Tangshen Formula (TSF)

The Tangshen formula (TSF), a herbal medicine from China, has exhibited encouraging effects in the management of NAFLD. Research indicates that TSF reduces hepatic steatosis and enhances lipid metabolism in several animal models [254,255,256]. Its mechanism of action involves various pathways, such as the modulation of gut microbiota and metabolic profiles [254], the activation of autophagy via the AMPK/SIRT1 pathway [255], and the regulation of macrophage activation and their phenotypic changes [256]. TSF treatment has been found to decrease lipid accumulation, lessen inflammation, and enhance insulin resistance and the integrity of the intestinal barrier [254,255]. Collectively, these findings suggest that TSF is a promising therapeutic option for NAFLD, functioning as a modulator of gut microbiota and metabolic profiles while also affecting hepatic cellular processes to reduce steatosis and related metabolic issues.

#### 7.1.9. Curcumin

Curcumin, a natural polyphenol, appears to be a promising candidate for the treatment of NAFLD. Research indicates that curcumin supplementation can lower liver fat levels, enhance lipid profiles, and reduce insulin resistance in patients with NAFLD [257]. The beneficial effects of curcumin are linked to its properties as an antioxidant, anti-inflammatory agent, and its ability to prevent fat accumulation [258]. From a mechanistic standpoint, curcumin inhibits the O-GlcNAcylation pathway, promoting antioxidant responses in NASH mice [259]. Furthermore, curcumin improves mitochondrial function via the SIRT3 pathway, reducing liver fat accumulation in postnatal overfed rats and fatty L02 cells [260]. These results imply that curcumin may represent an effective therapeutic option for NAFLD, targeting various components of the disease’s underlying mechanisms. Nevertheless, despite substantial experimental support, clinical evidence is still scarce, highlighting the necessity for more human studies to comprehensively determine curcumin’s effectiveness in treating NAFLD.

#### 7.1.10. Dihydromyricetin

Dihydromyricetin (DHM) has demonstrated encouraging effects in the treatment of NAFLD. Research suggests that DHM improves NAFLD by managing lipid and glucose metabolism, diminishing inflammation, and restoring the balance of gut microbiota [261,262]. DHM inhibits inflammatory signalling pathways, specifically TLR4/NF-κB, while promoting the growth of beneficial gut bacteria [262]. Additionally, it boosts mitochondrial function and redox equilibrium through SIRT3-dependent pathways, enhancing both mitochondrial respiratory capacity and antioxidant functions [262,263]. Laboratory studies indicate that DHM shields hepatocytes from lipid build-up and oxidative stress induced by oleic acid by inhibiting lipogenesis and modulating the PPARγ, AMPK, and AKT signalling pathways [264]. These results imply that DHM has the potential to serve as a therapeutic option for NAFLD, targeting various factors involved in the disease’s development, such as lipid metabolism, inflammation, oxidative stress, and gut microbiota imbalance.

### 7.2. Sirtuin Inhibitors

In the realm of hepatocellular carcinoma (HCC), the pharmaceutical sector has investigated the potential of sirtuin-targeting compounds, including the SIRT2 inhibitor salermide. This compound has demonstrated anti-tumour effects in preclinical models using human liver cancer cell lines, specifically HepG2 and Hep3B. Salermide inhibits SIRT2-mediated deacetylation of key metabolic enzymes, disrupting energy metabolism and reducing cell invasion [195].

AGK-2, a SIRT2 inhibitor, demonstrates an improvement in liver fibrosis induced by D-galactose in a rat model. This effect comes from its ability to inhibit fibrogenic factors, reduce inflammation, and decrease SIRT2 protein expression, suggesting a protective role against liver injury [265]. One study suggests that AK-7, another SIRT2 inhibitor, enhances p53 activity by preventing its deacetylation in non-small cell lung cancer (NSCLC) cells, positioning it as a potential candidate for cancer treatment, particularly for targeting p53-proficient cancers [266]. However, the specific role of AGK-2 and AK-7 in HCC needs further research. Overall, the therapeutic potential of SIRT2 inhibition remains contentious, with some studies suggesting both tumour-promoting and tumour-suppressing effects [267,268].

### 7.3. Gene Editing Approaches

Gene editing technologies like CRISPR/Cas9 provide innovative strategies to modulate sirtuin genes in HCC. This technology has shown potential in targeting cancer cells and creating engineered T cells for HCC treatment [269]. Additionally, CRISPR/Cas9 can alter the immunosuppressive environment in HCC by targeting genes such as GDF15 [270]. Targeting SIRT1 or combining conventional chemotherapy with SIRT1 inhibitors may improve therapeutic efficacy in HCC [43]. Overall, CRISPR/Cas9 and sirtuin modulation are promising strategies for treating HCC [271,272].

### 7.4. Small Molecule Targeting

Another strategy involves developing small-molecule inhibitors that target sirtuins. For instance, Binarci et al. have created novel indole-based compounds that effectively inhibit sirtuin activity in HCC cell lines, showing similar effectiveness to established inhibitors like EX-527 [273,274]. This suggests that these small molecules could serve as valuable therapeutic agents in HCC. Furthermore, the use of proteolysis-targeting chimeras (PROTACs) to degrade SIRT6 has shown promise, significantly reducing SIRT6 levels and inhibiting HCC cell proliferation [275]. This innovative method may offer a more precise way to target sirtuin activity compared to traditional inhibitors.

## 8. Gut Microbiota-Based Interventions: Probiotics, Prebiotics, and Synbiotics

Alongside sirtuin-targeted therapies, the pharmaceutical field is exploring gut microbiota-based interventions for disease management. Strategies addressing dysbiosis encompass FMT, probiotics, prebiotics, and dietary modifications [276]. Probiotics are beneficial live microorganisms, whereas prebiotics are selective substrates for host microorganisms [277]. These components can influence gut microbiota, improve immune response, and alleviate various health conditions, including gastrointestinal disorders, allergies, and infections [278]. The combined application of probiotics and prebiotics, referred to as synbiotics, demonstrates positive effects on the maintenance of a healthy gut microbiome [279]. Probiotics, prebiotics, and synbiotics have exhibited effectiveness in modulating immune responses, treating infections, managing inflammatory bowel diseases, and augmenting cancer treatment modalities [280].

**Figure 5:**
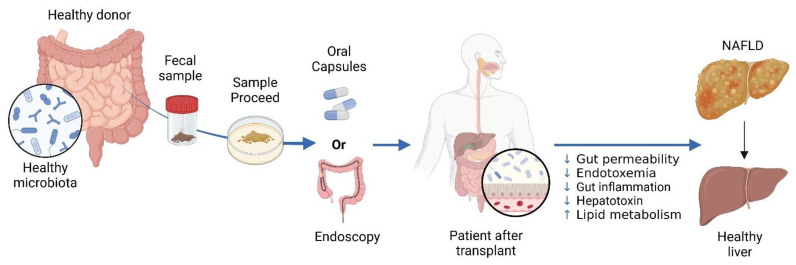
The Use of Fecal Microbiota Transplantation (FMT) in Treating NAFLD. In FMT, a healthy donor’s stool is processed and administered to a patient with NAFLD. Delivery can be achieved through oral or endoscopic methods. NAFLD is linked to gut dysbiosis and heightened intestinal permeability, which facilitates the transfer of gut-derived factors to the liver, exacerbating the condition. FMT aims to rectify dysbiosis and enhance the gut barrier, with the goal of improving liver health in individuals with NAFLD. Created in BioRender. https://BioRender.com/r59u349 (accessed on 12 March 2025).

In NAFLD, FMT is a novel strategy to modify gut microbiome and achieve metabolic balance [103]. FMT entails transferring a healthy microbiome to individuals with dysbiosis. The main aim is to replenish the recipient’s gut with beneficial microbes and restore a balanced microbial population [281]. FMT has shown promise in improving intestinal structure and function, enhancing lipid metabolism, reducing insulin resistance, suppressing inflammation, and alleviating NAFLD symptoms [282,283]. (see Figure 5).

By diversifying sirtuin-targeting and gut microbiome-modulating interventions, the pharmaceutical industry seeks to offer enhanced and individualised treatment modalities for various diseases, capitalising on the intricate interactions between these biological pathways and their health ramifications.

## 9. Future Directions

Future research should concentrate on elucidating the specific mechanisms by which sirtuins influence gut microbiota composition and function, particularly in the context of metabolic disorders and cancer. It is crucial to explore how different sirtuin isoforms modulate gut health and contribute to the progression of non-alcoholic fatty liver disease (NAFLD) and its transition to hepatocellular carcinoma (HCC). Clinical trials assessing the efficacy of selective sirtuin activators or inhibitors in treating metabolic dysfunctions and cancer are essential, as they could provide valuable insights into how targeting sirtuin pathways may benefit patients with NAFLD or liver cancer.

Additionally, investigating the role of gut microbiota-derived metabolites in regulating sirtuin activity could inform therapeutic strategies. Understanding the microbiome profiles associated with these conditions will be vital for developing personalised treatment approaches. Moreover, examining the interplay between sirtuins, gut microbiota, and immune responses in clinical settings may uncover novel approaches to enhance the effectiveness of immunotherapies for liver cancer.

This focused research direction has the potential to advance the understanding of sirtuin–microbiota interactions, ultimately leading to innovative therapies aimed at preventing and treating metabolic disorders and liver cancer.

## 10. Conclusions

The intricate interplay between sirtuins and gut microbiota is a crucial hub for regulating metabolic health and disease. Sirtuins significantly influence both the composition and functionality of gut microbiota while maintaining the integrity of the intestinal barrier and modulating immune responses. In turn, metabolites produced by gut microbiota can affect sirtuin activity, creating a dynamic feedback loop that impacts overall health.

Given the rising prevalence of non-alcoholic fatty liver disease (NAFLD) and its progression to hepatocellular carcinoma (HCC), understanding the molecular mechanisms underlying these interactions is essential for developing targeted therapeutic interventions. However, the composition of the gut microbiome differs greatly among people, calling for individualised treatment strategies. Although the prospects for personalised treatments based on the microbiome are promising, there are still hurdles to overcome, including enhancing the understanding of the mechanisms, standardising testing methods, and confirming results in larger cohorts. This review highlights the promise of novel strategies that modulate sirtuin and microbiota pathways to improve health outcomes. It emphasises the urgent need for further research into the detailed mechanisms of sirtuin-microbiota interactions and their implications for preventing and treating metabolic disorders and liver cancer. Although there is potential, additional studies are necessary to thoroughly comprehend these complex interactions and create successful interventions, such as tailored microbiome therapies and engineered microbial communities.

## Figures and Tables

**Table 1:** Classification and functional roles of sirtuins in metabolism and cellular processes.

Sirtuin	Class	Type of Activity	Acyl Substrates	Cellular Function	Target Substrates	Metabolic Role	Biological Role	References
**SIRT1**	I	Deacetylase	Remove acetyl and long chain fatty acyl group from Lysine	Chromatin structure development, mitochondrial biogenesis	NF-κB, p53, FOXO1, FOXO3, TORC2, PGC-1α, PPAR-γ, SREBP, LXR, FXR, LKB1	Fatty acid oxidation, cholesterol and bile acid homeostasis	Cell survival and metabolism	[44,45]
**SIRT2**	I	Deacetylase	Remove of acetyl, long-chain fatty acyl, 4-oxononanoyl, and benzoyl groups	Neurodegeneration, cell cycle control, cell motility	α-Tubulin, p53, p300, NF-κB FOXO1, FOXO3, HIF1α, PEPCK	Lipid metabolism, glucose homeostasis	Cell cycle regulation, tumour suppression/promotion, metabolism	[46,47,48,49]
**SIRT3**	I	Deacetylase	Remove acetyl and long-chain fatty acyl groups from lysine	Protection against oxidative stress, regulation of mitochondrial function and metabolism, ATP production	IDH2, LCAD, AceCS2, MnSOD, Ku70, HMGCS2, OTC, subunits of the ETC (complexes I–III and ATP synthase)	Fatty acid oxidation, amino acid metabolism, urea cycle promotion, and ketone body formation	Thermogenesis, oxidative stress resistance, tumour suppression	[50,51,52,53,54,55]
**SIRT4**	Class II	Mono-ADP-ribosyl transferase activity, Deacetylase, Lipoamidase	Remove lipoyl, biotinyl, hydroxymethylglutaryl, 3-methylglutaryl and 3-methylglutaconyl groups	Regulation of mitochondrial metabolism	GDH, MCD, MTP-α, PDH, MCCC, ANT2, ANT3, IDE	Glucose metabolism, fatty acid oxidation, amino acid catabolism	Insulin secretion, metabolic homeostasis, tumour suppression	[56,57,58]
**SIRT5**	Class III	Deacetylase, Desuccinylase, Deglutarylase, Demalonylase	Remove succinyl, glutaryl, and malonyl groups	Regulation of mitochondrial metabolism and ammonia detoxification	CPS1, GLUD1, UOX, GDH, IDH2, SDHA	Urea cycle and TCA cycle regulation, fatty acid and amino acid metabolism	Cellular energy homeostasis, and metabolism	[59,60,61,62]
**SIRT6**	Class IV	Deacetylase, Mono-ADP-ribosyl transferase activity	Remove acetyl and long-chain fatty acyl groups	DNA repair, telomeric preservation	PARP1, TNFα, NF-κB, GCN5, PPARα HIF1α TRF2	Regulation of glucose and lipid metabolism	Genomic stability, glucose homeostasis, inflammation control	[63,64,65]
**SIRT7**	Class IV	Deacetylase	Remove acetyl groups	rDNA transcription, ribosome biogenesis, cell proliferation, DNA repair, cellular senescence	RNA Pol I, PAF53, U3–55k, GABPβ1, H3K18, H3K122, NPM1	Lipid metabolism	Cell cycle regulation, tumour promotion, ageing, metabolic homeostasis	[66,67,68]

**Table 2:** Roles of sirtuins in gut health: impact on barrier integrity, inflammation, and microbial diversity.

Sirtuin	Roles of Sirtuins in Gut Health	References
SIRT1	Maintains intestinal epithelial barrier integrity, regulates inflammation, and modulates autophagy, potentially influencing gut microbiota composition and diversity.	[107,119,120,121]
SIRT2	Regulates intestinal epithelial cell proliferation and differentiation, impacting the gut environment and reducing inflammation, facilitating better host-microbiota interactions.	[111,122,123]
SIRT3	Enhances mitochondrial function in intestinal cells, regulates oxidative stress, and maintains gut barrier homeostasis; deficiency leads to microbial dysbiosis and impaired permeability.	[110,113,124]
SIRT4	Modulates amino acid metabolism in intestinal cells, potentially influencing nutrient availability for gut microbiota.	[115,125]
SIRT5	Regulates cellular homeostasis and various metabolic pathways in intestinal cells, potentially influencing nutrient availability for gut microbiota.	[60,126]
SIRT6	Maintains intestinal epithelial barrier integrity, mitigates inflammation, and enhances favourable immune responses; may affect gut microbiota composition and diversity.	[127,128,129]
SIRT7	Maintains intestinal homeostasis and modulates inflammation; potentially affecting gut microbiota composition.	[118,130]

## Data Availability

No new data were created.
